# Validation and potential refinement of the Malaysia Medication Adherence Assessment Tool (MyMAAT) among patients with non-dialysis chronic kidney disease

**DOI:** 10.1371/journal.pone.0332236

**Published:** 2025-09-26

**Authors:** Ming Chiang Lim, Ernieda Hatah, Jia Ean Goh, Ahmad Ramdani Abd Manan, Joyce Chu Yee Tan

**Affiliations:** 1 Department of Pharmacy, Hospital Sultan Haji Ahmad Shah, Ministry of Health Malaysia, Temerloh, Pahang, Malaysia; 2 Centre for Quality Management of Medicines, Faculty of Pharmacy, Universiti Kebangsaan Malaysia, Kuala Lumpur, Malaysia; 3 Pharmacy Unit, Klinik Kesihatan Triang, Ministry of Health Malaysia, Triang, Pahang, Malaysia; UCSI University, MALAYSIA

## Abstract

**Background:**

Medication adherence is essential for managing chronic kidney disease (CKD) and preventing its progression. In Malaysia, adherence is commonly assessed using the 12-item Likert scale Malaysia Medication Adherence Assessment Tool (MyMAAT-12), but it has not been validated for CKD patients. Thus, this study aimed to validate and explore its potential for item reduction among non-dialysis CKD patients.

**Methods and findings:**

A cross-sectional, multi-center study was conducted across eight health facilities in Pahang state from December 2023 to August 2024. Non-dialysis CKD patients aged 18 years and above, with or without diabetes, attending outpatient clinics, were given the 12-item self-administered MyMAAT-12 and items from the Beliefs about Medicines Questionnaire (BMQ). Patient demographics, HbA1c levels, and serum phosphate changes within three months preceding MyMAAT-12 completion were collected.

The dimensionality of the scale was explored through exploratory factor analysis (EFA), and its reliability and criterion validity were evaluated to ensure robust measurement properties. A simulation test was conducted to explore the feasibility of reducing MyMAAT-12 to a shorter version.

A total of 246 respondents participated, predominantly female (54.5%) and Malay (91.5%), with a mean age of 59.5 ± 12.5 years and an average CKD duration of 5.0 ± 5.4 years. The average medication adherence score was poor to moderate (<54/60). MyMAAT-12 demonstrated excellent internal consistency (Cronbach’s alpha = 0.90) and significant association with BMQ categories (χ²(2) = 16.8, p < 0.01). EFA identified a two-factor structure. MyMAAT-12 adherence categorization was significantly associated with HbA1c% (<8% vs. ≥ 8%) (χ^2^(2) = 4.7, p = 0.03) and phosphate level changes (χ^2^(2) = 5.48, p = 0.02). Results indicated that reducing MyMAAT-12 to a 7-item scale was feasible, while maintaining strong psychometric properties (Cronbach’s alpha = 0.87) and a significant association with HbA1c% (ρ = −0.22, p = 0.01).

**Conclusion:**

MyMAAT-12 demonstrated strong reliability and validity, making it a valuable tool for clinicians to accurately assess medication adherence in non-dialysis CKD patients. The shortened MyMAAT-7 offers a streamlined alternative with strong psychometric properties, enhancing clinical practicality.

## Introduction

Chronic kidney disease (CKD) is a chronic condition characterized by kidney damage or reduced kidney function, indicated by an estimated glomerular filtration rate (eGFR) of less than 60 ml/min/1.73m^2^ for at least three months, irrespective of the cause [1]. It is a major public health issue in Malaysia, with its prevalence rising from 9.1% in 2011 to 15.5% in 2018 [2]. The Malaysian Dialysis and Transplant Registry reported a growing number of patients with CKD progressing to end-stage kidney disease (ESKD), increasing from 29,452 patients in 2012–51,256 patients in 2022 [3]. The number of patients with ESKD is predicted to reach 106,000 in 2040 if current trends persist, incurring a 3.2 billion Ringgit Malaysia burden on the healthcare system [4].

Patients with CKD, regardless of whether they undergo renal replacement therapy, often present with multiple comorbidities such as diabetes mellitus, hypertension, obesity, atherosclerotic vascular disease, and metabolic syndrome [1]. When identified early and managed appropriately, the decline in kidney function can be reduced by up to 50% [5], and adherence to prescribed medications plays a critical role in achieving this. Medication adherence is defined as the extent to which a person’s behaviour conforms to the recommendations of healthcare professionals [6]. It can be affected by various factors, including socioeconomic factors, the provider-patient relationship, disease severity, complexity of the treatment regimen, cultural beliefs, and perceived value of medications [6–8]. In CKD specifically, adherence is frequently suboptimal due to complex treatment regimens involving multiple medications and a high pill burden. A systematic review found medication adherence rates among CKD patients ranged from 33% to 88% across 54,652 individuals [9]. A recent study in Malaysia also revealed that up to 70% of patients with CKD poorly adhered to their medications, especially when prescribed more than seven medications daily [10]. Additionally, patients in the later stages of CKD were found to experience higher levels of anxiety and stress, further compromising adherence and disease control. These findings underscore the importance of early interventions to improve adherence and reduce disease progression [9,11].

Given the critical impact of adherence on disease outcomes, research in this area is increasingly important. Various methods are available to measure medication adherence, including both subjective and objective approaches. Objective methods include direct measurements of clinical outcomes, such as biomarkers, pharmacy refill records, and drug concentration levels. In contrast, subjective methods typically involve self-report questionnaires or interviews with patients or their caregivers [9]. Although numerous tools exist to evaluate medication adherence, each has its own limitations. For instance, objective assessments may be inaccurate for medications with variable pharmacokinetics, drugs that do not have measurable serum levels, or in cases where clinical outcomes are influenced by multiple confounding factors. Meanwhile, self-reported questionnaires are prone to social desirability bias and recall errors, which may compromise the accuracy of the results [9].

Among subjective assessments, one notable tool for measuring medication adherence is the Beliefs about Medicines Questionnaire (BMQ), developed based on the Necessity-Concern Framework (NCF) [12]. According to the NCF, patients weigh the “costs” and “benefits” of medication use and are more likely to adhere when they perceive the necessity of treatment to outweigh their concerns [12–14]. The BMQ has been validated and found to correlate well with medication adherence across multiple conditions [15]. The Malaysia Medication Adherence Assessment Tool (MyMAAT) is a recently developed instrument tailored to the Malaysian population, originally designed for patients with diabetes while incorporating local perspectives and cultural considerations [16]. This 12-item tool measures beliefs, self-efficacy, and concerns about adverse effects related to medication adherence. Since its development, MyMAAT-12 has been widely used in Malaysian government healthcare settings and applied across diverse populations, including patients with tuberculosis, those at risk of cardiovascular diseases, and older adults with chronic conditions [17–21].

Although MyMAAT-12 has been widely used in public health facilities in Malaysia to assess medication adherence, its validity in patients with CKD remains unexplored – a population known to have complex medication regimens and high rates of nonadherence. Given these unique challenges, validating MyMAAT-12 in CKD patients is essential to ensure accurate assessment and guiding appropriate interventions. This study aims to bridge this gap by evaluating medication adherence in non-dialysis CKD patients and validating MyMAAT-12 for use in this group.

## Methods

### Study design and setting

This cross-sectional study was conducted at six public hospitals and two primary health clinics in Pahang, Malaysia, between 15^th^ December 2023 and 31^st^ August 2024. Facilities were selected based on the availability of a pharmacy electronic system capable of accessing and retrieving patients’ medical records.

### Inclusion and exclusion criteria

Using convenience sampling, patients meeting the following criteria were invited to participate: adults aged 18 years or older, able to speak and understand English or Bahasa Malaysia, and diagnosed with secondary CKD stage 3–5 prior to the study. CKD stages were classified based on the glomerular filtration rate category estimated using the Chronic Kidney Disease Epidemiology Collaboration (CKD-EPI) Creatinine Equation 2021 [1,22]. Stage 3 CKD was defined as an eGFR of 30–60 ml/min/1.73m^2^, stage 4 as 15–29 ml/min/1.73m^2^, and stage 5 as <15 ml/min/1.73m^2^.

Patients were excluded if they had been on dialysis for at least three months or had recently started regular dialysis, were pregnant, had Alzheimer’s disease, dementia, severe psychopathology (e.g., schizophrenia, bipolar disorder), cognitive impairment, did not manage their own medications, or submitted an incomplete questionnaire with more than 20% missing responses. CKD patients with underlying conditions such as glomerulonephritis, polycystic kidney disease, congenital malformations, autoimmune diseases (e.g., lupus nephritis), kidney stones, or kidney cancer were also excluded, as these primary kidney diseases differ in clinical course and management from secondary causes, which are the main focus of this study [3].

### Data collection

Patients meeting the inclusion criteria were recruited from the outpatient pharmacy department, where the study was explained to them, and they were provided with study information. Those who agreed gave written consent form, completed a self-administered bilingual questionnaire (English and Malay), and granted access to their medical records for data collection. The questionnaires were completed on-site while patients waited for their medications, requiring approximately 15–20 minutes. No incentives were offered to participants. Among the respondents, 27 patients with short appointment intervals were randomly invited to participate and, upon agreeing, were asked to return to the pharmacy in two weeks to complete the questionnaire again for test-retest reliability.

### Sample size calculations

As both the MyMAAT-12 and BMQ scales had already been validated in their respective populations and settings, content validity and face validity tests were not repeated. For the validation of the 12-item algorithm among patients with CKD, a subject to item ratio of 20:1 for exploratory factor analysis (EFA) indicated a minimum sample size of 240 patients [23]. For test-retest reliability to determine agreement on the adherence score across two periods, the sample size was calculated based on an Intraclass Correlation Coefficient (ICC) of 0.5, an alpha of 0.05, and power of 80% [24]. This yielded a sample size of 22. Based on standard psychometric benchmarks, an ICC of 0.5 was selected as it reflects the minimum acceptable level of moderate reliability for newly developed or adapted instruments in health research [24]. An additional 20% was added for potential dropouts, bringing the total required sample to 27.

### Tools

The self-administered questionnaire comprised four main sections. Section A gathered patients’ demographic information, including age, gender, race, disease duration, and education level. Section B assessed medication adherence using the MyMAAT-12, a 12-item tool rated on a five-point Likert scale, ranging from “strongly agree” (1) to “strongly disagree” (5). The tool evaluated adherence across five conceptual domains: medication-taking behaviour; perceived utility of medications; perceived barriers to adherence; self-efficacy and social support; and other relevant behavioral influences [16]. The MyMAAT-12 demonstrated excellent internal consistency (Cronbach’s alpha = 0.91) and strong test-retest reliability (ICC = 0.97) in a diabetes patient population. A cut-off score of 54 classified patients with scores between 12 and 54 as having poor to moderate adherence, while scores between 55 and 60 indicated good adherence [16].

Section C estimated medication adherence and beliefs using BMQ. The BMQ consisted of two major sections: general and specific. The general section contained eight questions that gauged broad beliefs about medicines using the general-overuse scale and general-harm scale [25]. Meanwhile, the specific section utilized the NCF, with patients responding to 10 questions specific to their disease [12]. Our study employed the BMQ-Specific scale as a tool for concurrent validity, which has been validated among Chinese patients with CKD not undergoing dialysis and demonstrated concurrent validity with the Morisky Medication Adherence Scale [26].

The BMQ scale comprises two five-item subscales assessing patients’ beliefs about the necessity of medications for managing chronic disease and concerns over adverse effects [26]. Responses were scored on a five-point Likert scale from 1 (“strongly disagree”) to 5 (“strongly agree”), with subscale scores ranging from 5 to 25. Following the general reported responses classification, the responses were classified into four groups: 1) accepting (Necessity scores ≥15 and Concern scores <15); 2) ambivalent (Necessity scores ≥15 and Concern scores ≥15); 3) indifferent (Necessity scores <15 and Concern scores <15); and 4) skeptical (Necessity scores <15 and Concern scores ≥15) [12,13,26]. Previous research indicated that medication adherence was highest in the accepting group, followed by the ambivalent, indifferent, and skeptical groups [12]. A necessity-concern differential score (ranging from −20–20) was calculated by subtracting the concern subscale score from the necessity subscale score, accounting for the different dimensions of necessity and concern. A higher differential score implied stronger beliefs in the necessity for medication compared to concerns about adverse consequences. High adherence among CKD patients not on dialysis was proposed to correlate with high necessity and low concern [26]. Therefore, in the current study, participants in the accepting category were classified as adherent, while all other categories were classified as non-adherent.

The MyMAAT-12 and BMQ-specific scales were available in Malay and English as priorly translated and validated, allowing patients to choose the language they preferred [16,27,28]. Three recent laboratory parameters prior to the survey completion of the patients, including serum creatinine, serum phosphate, and hemoglobin A1c (HbA1c) levels were collected by the site investigators who were also pharmacists.

### Data analysis

Data analysis was conducted using Statistical Package for the Social Science (SPSS) software version 24. Categorical demographic data was presented as frequencies and percentages, with statistical significance set at a p-value of less than 0.05 for all inferential tests. To analyze the relationship between respondents’ MyMAAT-12 and BMQ scores and demographic data, Mann-Whitney U tests were used to assess association of scores with gender and number of medications, while the Kruskal-Wallis test was used to associate scores with duration of CKD diagnosis and CKD stages.

The dimensionality of the scale was measured using EFA to test the underlying structures among variables and provide evidence of unidimensionality across items. Factorability was confirmed using the Kaiser-Meyer-Olkin (KMO) measure of sampling adequacy (target ≥ 0.70) and Bartlett’s test of sphericity (p < 0.05). Oblique rotation with Direct Oblimin analysis was used due to potential correlations among items measuring the same construct. Inter-item correlations were targeted between 0.3 and 0.9. Additionally, the percentage of variance explained, or average variance extracted (AVE), was targeted at more than 50%. Item factor loadings and significance of factor loadings were evaluated to confirm unidimensionality, with a minimum acceptable factor loading of 0.40 (0.50 and above indicating strong loading), and a p-value of 0.05 indicated a significant factor loading for each item [29].

Internal consistency was tested using Cronbach’s alpha, with ideal correlation coefficients targeted between 0.60 and 0.90 [29]. Test-retest reliability was used to assess stability of measurements over time. It was assessed using ICC, applying a two-way mixed-effects model with absolute agreement, interpreted as poor (<0.40), fair to good (0.40–0.75), or excellent (≥0.75) [30].

Hypothesis testing with criterion validity test was utilized to measure the correlation between MyMAAT-12 scores and the BMQ-specific scale and clinical parameters (serum phosphate level changes and HbA1c%) using Spearman’s correlation test. MyMAAT-12 scores were calculated by assigning points to individual items as follows: five points for ‘strongly disagree’, four for ‘disagree’, three for ‘neutral’, two for ‘agree’, and one for ‘strongly agree’. The total MyMAAT-12 score was the sum of points across all items. Using a cut-off score of 54, patients were grouped into two groups: (1) good adherence and (2) poor to moderate adherence [16]. Validity against known groups was assessed using HbA1c categories of ≥8% and <8%, as stated in the Malaysian Diabetes Clinical Practice Guideline (2020) [31] and serum phosphate level trends (increasing trend or otherwise) using chi-square tests. Sensitivity, specificity, and positive and negative predictive values (precision rate) of the MyMAAT-12 were calculated against the BMQ score, HbA1c%, and serum phosphate level changes.

A simulation test was conducted to explore the feasibility of reducing the MyMAAT-12 scale. This approach sought to retain the key dimensions of adherence while improving usability. Evidence suggests reducing item numbers may alleviate response burden and improve engagement, particularly in clinical settings where patients may benefit from streamlined assessments [32]. Items with high communalities, strong factor loadings, and conceptual relevance were retained. To establish an optimal cutoff point for the reduced MyMAAT scale, Receiver Operating Characteristic (ROC) curve analysis was performed. This approach allowed for the evaluation of sensitivity and specificity across various threshold values, helping to identify the cutoff that maximizes the tool’s discriminative power between adherent and non-adherent groups. The sensitivity, specificity, and predictive values of the simulated MyMAAT scale were tested against the full MyMAAT-12, BMQ, HbA1c%, and serum phosphate level changes. The study obtained ethical approval from the Medical Research & Ethics Committee, National Institutes of Health Malaysia with reference number of NMRR-23–02445-G6O.

## Results

### Demographic characteristics

A total of 263 respondents participated in the study. However, 12 were excluded for not meeting the inclusion criteria (Stage 1 to Stage 2 CKD), three were excluded due to receiving dialysis treatment within the past three months, and two surveys were excluded due to incomplete responses. This resulted in a final sample of 246 patients for analysis. The majority of respondents were female (n = 134, 54.5%) and Malay (n = 225, 91.5%), with a mean age of 59.5 years (SD = 12.5). On average, patients had been diagnosed with CKD for five years (SD = 5.4) and were taking 7.6 ± 2.3 medications daily. The demographic characteristics of the respondents are summarized in Table 1.

**Table 1:** The respondents’ demographic characteristics (n = 246).

Demographic variables	Number (n)	Percentage (%)
**Age**		
< 65 years old	153	62.2
≥ 65 years old	93	37.8
**Gender**		
Male	112	45.5
Female	134	54.5
**Race**		
Malay	225	91.5
Chinese	13	5.3
Indian	6	2.4
Other	2	0.8
**Highest education level**		
No formal education	13	5.3
Primary education	54	22.0
Secondary education	147	59.8
Diploma	19	7.7
Bachelor’s degree	13	5.3
**Month Income**		
< RM 1,000	141	57.3
RM 1,000 – RM 3,000	73	29.7
RM 3,001 – RM 5,000	26	10.6
> RM 5,000	6	2.4
**Duration of CKD**		
< 5 years	149	60.6
5–10 years	72	29.3
> 10 years	25	10.2
**CKD Stages**		
CKD Stage 3 (30 < eGFR < 60 ml/min/1.73m^2^)	71	28.9
CKD Stage 4 (15 ≤ eGFR < 30 ml/min/1.73m^2^)	102	41.5
CKD Stage 5 (eGFR < 15 ml/min/1.73m^2^)	73	29.7
**Number of medications**		
≤ 5 medications	41	16.7
> 5 medications	205	83.3
**MyMAAT Medication Adherence Level**		
Poor to moderate	143	58.1
Good	103	41.9

### Medication belief and adherence

**Fig 1:**
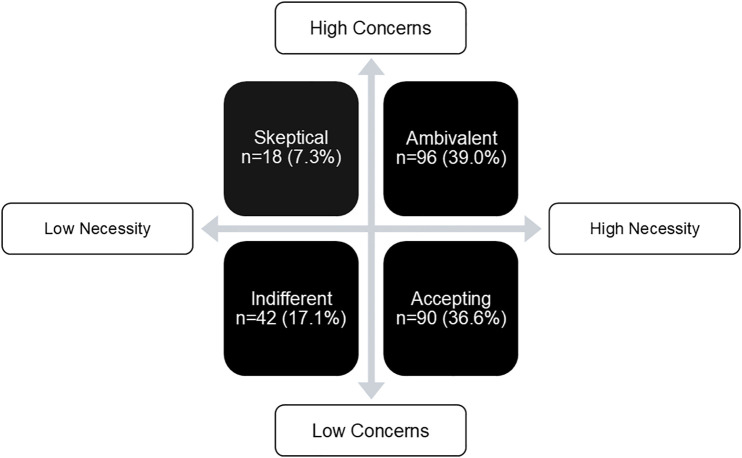
Distribution of the beliefs and attitudes towards medication (n = 246).

The mean MyMAAT-12 score among respondents was 50.7 (SD = 9.0), indicating poor to moderate medication adherence. The descriptive analysis of MyMAAT-12 scores is presented in Table 2. Adherence scores did not differ significantly by gender (p = 0.10), age group (p = 0.52), number of medications (p = 0.17), CKD stages (p = 0.05), or CKD duration (p = 0.18). Respondents demonstrated stronger beliefs in the necessity of their prescribed medications (mean BMQ-Necessity score: 17.9 ± 4.4) than concerns about adverse effects (mean BMQ-Concern score: 14.2 ± 4.4). The necessity-concern differential score was 3.7 ± 5.7, ranging from −13–20. While most respondents (n = 186, 75.6%) reported high perceived necessity, 46.3% also expressed high levels of concern. The scores for each BMQ statement are summarized in Table 3. Patients were categorized into four groups based on their Necessity and Concern scores, as illustrated in Fig 1. Similar to the MyMAAT-12 scores, BMQ-Necessity and BMQ-Concern scores did not vary significantly across gender (p = 0.12; p = 0.15), age group (p = 0.93; p = 0.29), number of medications (p = 0.15; p = 0.16), CKD stages (p = 0.58; p = 0.34), or CKD duration (p = 0.32; p = 0.94).

**Table 2:** Descriptive analysis of respondents’ MyMAAT-12 scores (n = 246).

Domains	Variables	Strongly Disagree	Disagree	Neutral	Agree	Strongly Agree
Medication taking behaviour	1. In the past one month, I frequently failed to take my medication in accordance with the doctor’s instruction.	131 (53.3%)	69 (28.0%)	10 (4.1%)	29 (11.8%)	7 (2.8%)
	2. In the past one month, I reduced my medication intake when I felt better.	136 (55.3%)	77 (31.3%)	6 (2.4%)	19 (7.7%)	8 (3.3%)
	3. In the past one month, I took my medication alternately.	143 (58.1%)	73 (29.7%)	10 (4.1%)	14 (5.7%)	6 (2.4%)
	4. I was often late on/ missed the appointment date to get the supplies of my follow-up medication at the pharmacy counter.	122 (49.6%)	86 (35.0%)	10 (4.1%)	23 (9.3%)	5 (2.0%)
Others	5. I have excess supply of the prescribed medication at home.	97 (39.4%)	105 (42.7%)	11 (4.5%)	22 (8.9%)	11 (4.5%)
Perceived utility	6. I did not fully comply with the prescriptions because I felt it was unnecessary/insignificant	138 (56.1%)	79 (32.1%)	7 (2.9%)	15 (6.1%)	7 (2.8%)
Perceived barriers	7. In the past one month, I frequently failed to remember to take my medication.	122 (49.6%)	91 (37.0%)	9 (3.6%)	19 (7.7%)	5 (2.0%)
	8. I regularly take less medication than prescribed for fear of the side effects to my body.	117 (47.6%)	84 (34.1%)	4 (1.6%)	27 (11.0%)	14 (5.7%)
Sociocognitive theory	9. I will miss/not take my medication if no one reminds me to do so.	143 (58.1%)	68 (27.6%)	12 (4.9%)	12 (4.9%)	11 (4.5%)
	10. I am uncertain about my daily medication doses.	140 (56.9%)	72 (29.3%)	12 (4.9%)	11 (4.5%)	11 (4.5%)
	11. I am unable to manage my medication intake properly.	128 (52.0%)	79 (32.1%)	12 (4.9%)	14 (5.7%)	13 (5.3%)
	12. Without support or help from the loved ones, I lack motivation to take my medication as prescribed by the doctor.	139 (56.5%)	63 (25.6%)	8 (3.3%)	20 (8.1%)	16 (6.5%)

**Table 3:** The respondents’ belief on medication (n = 246).

Domain	Variables	Strongly Disagree	Disagree	Neutral	Agree	Strongly Agree
Necessity	1. My health, at present, depends on these medicines	8 (3.3%)	38 (15.4%)	35 (14.2%)	89 (36.2%)	76 (30.9%)
Concerns	2. Having to take medicines worries me	40 (16.3%)	72 (29.3%)	42 (17.1%)	67 (27.2%)	25 (10.2%)
Necessity	3. My life would be impossible without these medicines	20 (8.1%)	50 (20.3%)	64 (26.0%)	74 (30.1%)	38 (15.4%)
Necessity	4. Without these medicines I would be very ill	19 (7.7%)	37 (15.0%)	48 (19.5%)	90 (36.6%)	52 (21.1%)
Concerns	5. I sometimes worry about long-term effects of these medicines	28 (11.4%)	43 (17.5%)	43 (17.5%)	96 (39.0%)	36 (14.6%)
Concerns	6. These medicines are a mystery to me	36 (14.6%)	46 (18.7%)	110 (44.7%)	38 (15.4%)	16 (6.5%)
Necessity	7. My health in the future will depend on these medicines	15 (6.1%)	29 (11.8%)	54 (22.0%)	105 (42.7%)	43 (17.5%)
Concerns	8. These medicines disrupt my life	69 (28.0%)	86 (35.0%)	47 (19.1%)	27 (11.0%)	17 (6.9%)
Concerns	9. I sometimes worry about becoming too dependent on these medicines	36 (14.6%)	69 (28.0%)	32 (13.0%)	79 (32.1%)	30 (12.2%)
Necessity	10. These medicines protect me from becoming worse	13 (5.3%)	35 (14.2%)	19 (7.7%)	103 (41.9%)	76 (30.9%)

Nevertheless, the BMQ-Concern scores differed significantly by adherence level (U = 5446, p < 0.01), with patients demonstrating good adherence scoring lower (12.0 ± 7.0) than those with poor to moderate adherence (15.0 ± 6.0). No significant differences were found between adherence level and BMQ-Necessity scores (p = 0.13). Moreover, the MyMAAT-12 score also varied significantly across the four necessity-concern differential groups (χ²(2) = 16.8, p < 0.01), with the accepting group reporting the highest adherence (55.0 ± 12.0), followed by indifferent (54.0 ± 9.0), ambivalent (48.0 ± 13.0), and skeptical (44.5 ± 22.0) groups.

### Psychometric properties of MyMAAT-12 scale

EFA was conducted to identify the smallest number of factors that best represented the scale’s items. Correlation matrix between items ranged from 0.26 to 0.72, Bartlett’s Test of Sphericity was significant (p < 0.01), and the KMO value was 0.90, indicating the scale was fit for factor analysis. Principal Axis Factoring revealed an AVE of 54.3%, with two factors extracted. Most items had communalities above 0.5, except item 4 (0.32), item 5 (0.38), and item 8 (0.44).

Using Oblique rotation with Direct Oblimin analysis, items 1–8 were loaded into factor 1; while items 9–12 were loaded into factor 2 with no items with factor loading of less than 0.40 The factor structure obtained is summarized in Table 4. These factors represented two dimensions: specific medication-taking behaviour (factor 1) and reasons for medication non-adherence related to self-efficacy and social support (factor 2), in line with Sociocognitive theory.

**Table 4:** Factor analysis of MyMAAT-12 scale (Direct Oblimin Rotated Component Matrix).

Items	Factor 1 Rotated Component Loading	Factor 2 Rotated Component Loading
Eigenvalue	5.89	1.46
% of variance explained	49.08	12.19
Cumulative %	61.27	
1. In the past one month, I frequently failed to take my medication in accordance with the doctor’s instruction.	0.78	
2. In the past one month, I reduced my medication intake when I felt better.	0.65	
3. In the past one month, I took my medication alternately.	0.74	
4. I was often late on/ missed the appointment date to get the supplies of my follow-up medication at the pharmacy counter.	0.56	
5. I have excess supply of the prescribed medication at home.	0.62	
6. I did not fully comply with the prescriptions because I felt it was unnecessary/insignificant	0.71	
7. In the past one month, I frequently failed to remember to take my medication.	0.78	
8. I regularly take less medication than prescribed for fear of the side effects to my body.	0.46	
9. I will miss/not take my medication if no one reminds me to do so.		−0.73
10. I am uncertain about my daily medication doses.		−0.81
11. I am unable to manage my medication intake properly.		−0.83
12. Without support or help from the loved ones, I lack motivation to take my medication as prescribed by the doctor.		−0.87

### Reliability of MyMAAT-12 scale among patients with CKD

The MyMAAT-12 demonstrated excellent internal consistency (Cronbach’s α = 0.90), with no item deletion improving the alpha (Table 5). Corrected Item-Total Correlation (CITC) coefficients showed moderate to strong correlations across all 12 items, ranging from 0.50 to 0.69. Test-retest reliability, conducted with 27 patients, yielded an ICC of 0.85 (95% CI: 0.69 to 0.93).

**Table 5:** Reliability analysis of MyMAAT-12 scale.

Items	Corrected Item-Total Correlations	Cronbach’s alpha if item deleted
1. In the past one month, I frequently failed to take my medication in accordance with the doctor’s instruction.	0.614	0.897
2. In the past one month, I reduced my medication intake when I felt better.	0.645	0.895
3. In the past one month, I took my medication alternately.	0.634	0.896
4. I was often late on/ missed the appointment date to get the supplies of my follow-up medication at the pharmacy counter.	0.502	0.902
5. I have excess supply of the prescribed medication at home.	0.552	0.900
6. I did not fully comply with the prescriptions because I felt it was unnecessary/insignificant	0.657	0.895
7. In the past one month, I frequently failed to remember to take my medication.	0.691	0.894
8. I regularly take less medication than prescribed for fear of the side effects to my body.	0.635	0.896
9. I will miss/not take my medication if no one reminds me to do so.	0.668	0.894
10. I am uncertain about my daily medication doses.	0.639	0.896
11. I am unable to manage my medication intake properly.	0.669	0.894
12. Without support or help from the loved ones, I lack motivation to take my medication as prescribed by the doctor.	0.655	0.895

### Hypothesis testing validity of MyMAAT-12 scale

The MyMAAT-12 score showed a significant positive correlation with the necessity-concern differential score (ρ = 0.24, p < 0.01) and a significant negative correlation with the BMQ-Concern score (ρ = −0.31, p < 0.01), but not with the BMQ-Necessity score (ρ = 0.09, p = 0.17). The MyMAAT-12 score also showed a significant negative correlation with HbA1c% (ρ = −0.26, p < 0.01), but not phosphate level changes (ρ = −0.14, p = 0.09).

However, when adherence scores were categorized as good versus poor to moderate adherence, a significant association was observed between adherence categories and HbA1c% (<8% and ≥8%) (χ^2^(2) = 4.71, p = 0.03) as well as phosphate level changes (χ^2^(2) = 5.48, p = 0.02), as shown in Table 6.

**Table 6:** MyMAAT-12 categories’ known groups validity.

MyMAAT-12 categories	HbA1c% (n, %)		χ^2^(df)	p-value*
	<8%	≥8%		
Poor to moderate adherence	44 (55.7%)	35 (44.3%)	4.71 (2)	0.03
Good adherence	38 (74.5%)	13 (25.5%)		
**MyMAAT-12 categories**	**Phosphate level changes (n, %)**		**χ** ^**2**^ **(df)**	p-value*
	**Did not increase**	**Increases**		
Poor to moderate adherence	32 (36.4%)	56 (63.6%)	5.48 (2)	0.02
Good adherence	33 (55.9%)	26 (44.1%)		

*Pearson Chi-square test.

Referring to Table 7, the calculated sensitivity of the MyMAAT-12 when tested against BMQ-adherence level, HbA1c levels, and phosphate level changes was 49.5%, 72.9%, and 68.3%, respectively. The computed MyMAAT-12 specificity using BMQ-adherence level, HbA1c levels, and phosphate level changes were 36.8%, 46.3%, and 50.8%, respectively. The false positive rate (Type I error) when tested against BMQ-adherence level, HbA1c levels, and phosphate level changes were 63.2%, 53.7%, and 49.2%, whereas the false negative rate (Type II error) was 50.5%, 27.1%, and 31.7% respectively. The positive predictive value (PPV) and negative predictive value (NPV) were 31.5% and 55.3% with BMQ-adherence level, 44.3% and 74.5% with HbA1c levels, and 63.6% and 55.9% with phosphate level changes.

**Table 7:** Sensitivity and specificity test for MyMAAT-12 scale.

MyMAAT-12 classification (n = 246)	BMQ (Ambivalent, Indifferent, and Skeptical)	BMQ (Accepting group)	Positive and negative predictive value
Poor to moderate adherence (Score <54)	45	98	Positive PV = 31.5%
Good adherence (Score ≥54)	46	57	Negative PV = 55.3%
Sensitivity and specificity	Sensitivity = 49.5%		Specificity = 36.8%
**MyMAAT-12 classification** **(n = 130)**	**Poor control HbA1c ≥ 8%**	**Good control HbA1c < 8%**	**Positive and negative predictive value**
Poor to moderate adherence (Score <54)	35	44	Positive PV = 44.3%
Good adherence (Score ≥54)	13	38	Negative PV = 74.5%
Sensitivity and specificity	Sensitivity = 72.9%		Specificity = 46.3%
**MyMAAT-12 classification** **(n = 147)**	**Phosphate level increases**	**Phosphate level did not increase (maintained/ reduced)**	**Positive and negative predictive value**
Poor to moderate adherence (Score <54)	56	32	Positive PV = 63.6%
Good adherence (Score ≥54)	26	33	Negative PV = 55.9%
Sensitivity and specificity	Sensitivity = 68.3%		Specificity = 50.8%

### Reduction analysis of MyMAAT-12

The study further explored the possibility of reducing the MyMAAT-12 scale. Guided by EFA findings, simulations were conducted to remove items with lower extracted communalities, specifically item 4 (0.32), item 5 (0.38), and item 8 (0.44). Additionally, items 1 and 12 were excluded following expert consensus and the high correlations of item 12 with item 11 (r = 0.68) and item 9 (r = 0.72).

EFA was then repeated for the revised 7-item scale (MyMAAT-7). Results indicated that reducing the original MyMAAT-12 to a 7-item scale was feasible, yielding a more streamlined tool without significant loss of explanatory power. The correlation matrix between items ranged from 0.33 to 0.72, Bartlett’s Test of Sphericity remained significant (p < 0.01), and KMO was 0.83, indicating suitability for factor analysis.

Principal Axis Factoring revealed two factors with a total variance explained of 70.9% and AVE of 60.2. All items had communalities > 0.5. Items 2, 3, 6, and 7 loaded onto Factor 1, while items 9, 10, and 11 loaded onto Factor 2. These factors were similar to the original MyMAAT-12. The lowest factor loading, 0.62, was observed for item 9, which remained within the acceptable range. The factor structure obtained is summarized in Table 8.

**Table 8:** Factor analysis of reduced item (simulated MyMAAT-7 scale).

Items	Factor 1 Rotated Component Loading	Factor 2 Rotated Component Loading
Eigenvalue	3.87	1.10
% of variance explained	55.27	15.71
Cumulative %	70.98	
2. In the past one month, I reduced my medication intake when I felt better.	0.75	
3. In the past one month, I took my medication alternately.	0.83	
6. I did not fully comply with the prescriptions because I felt it was unnecessary/insignificant	0.62	
7. In the past one month, I frequently failed to remember to take my medication.	0.74	
9. I will miss/not take my medication if no one reminds me to do so.		−0.62
10. I am uncertain about my daily medication doses.		−0.82
11. I am unable to manage my medication intake properly.		−0.92

### Reliability of MyMAAT-7 scale among patients with CKD

The MyMAAT-7 demonstrated an acceptable internal consistency (Cronbach’s alpha = 0.87), with factors aligning closely with the original MyMAAT-12 structure, suggesting that the shortened scale preserves the validity of the original measure while offering a more user-friendly format for respondents. Cronbach’s alpha would not have increased with the deletion of any items (Table 9). The CITC coefficients revealed moderate to strong correlations of all 7 items to the total scale, ranging from 0.61 to 0.67.

**Table 9:** Reliability analysis of simulated MyMAAT-7 scale.

Items	Corrected Item-Total Correlations	Cronbach’s alpha if item deleted
2. In the past one month, I reduced my medication intake when I felt better.	0.665	0.842
3. In the past one month, I took my medication alternately.	0.610	0.849
6. I did not fully comply with the prescriptions because I felt it was unnecessary/insignificant	0.620	0.848
7. In the past one month, I frequently failed to remember to take my medication.	0.621	0.848
9. I will miss/not take my medication if no one reminds me to do so.	0.639	0.845
10. I am uncertain about my daily medication doses.	0.642	0.845
11. I am unable to manage my medication intake properly.	0.656	0.843

### Hypothesis testing validity of MyMAAT-7 scale

Following the EFA, a ROC curve analysis was conducted to establish an optimal cutoff point for the MYMAAT-7. The ROC analysis evaluated the sensitivity and specificity of various threshold values, with the area under the curve of 0.99, indicating the scale’s discriminative accuracy. A cutoff score of 31.5 (out of 35) was suggested, providing a strong balance between sensitivity (99.0%) and a false positive rate of 15.4% when compared to the MyMAAT-12. With this, respondents scoring ≥32 were classified as having good adherence; those scoring <32 were categorized as having poor to moderate adherence.

Similar to MyMAAT-12, the MyMAAT-7 was positively correlated with the necessity-concern differential (ρ = 0.23, p < 0.01), negatively correlated with BMQ-Concern (ρ = −0.31, p < 0.01), and showed no significant correlation with BMQ-Necessity (ρ = 0.08, p = 0.21). A strong positive correlation was observed between MyMAAT-7 and MyMAAT-12 scores (ρ = 0.96, p < 0.01). The MyMAAT-7 score showed significant, negative correlations with HbA1c% (ρ = −0.22, p = 0.01) and phosphate level changes (ρ = −0.17, p = 0.04).

When adherence scores were categorized as good versus poor to moderate adherence, a significant association was observed between adherence categories and HbA1c% categories (<8% and ≥8%) (χ^2^(2) = 4.19, p = 0.04), but not with phosphate level changes (χ^2^(2) = 1.10, p = 0.29), as presented in Table 10.

**Table 10:** MyMAAT-7 categories’ known groups validity.

MyMAAT-7 categories	HbA1c% (n, %)		χ^2^(df)	p-value*
	<8%	≥8%		
Poor to moderate adherence	36 (54.5%)	30 (45.5%)	4.19 (2)	0.04
Good adherence	46 (71.9%)	18 (28.1%)		
**MyMAAT-7 categories**	**Phosphate level changes (n, %)**		**χ** ^**2**^ **(df)**	p-value*
	**Did not increase**	**Increases**		
Poor to moderate adherence	30 (40.0%)	45 (60.0%)	1.10 (2)	0.29
Good adherence	35 (48.6%)	37 (51.4%)		

*Pearson Chi-square test.

Referring to Table 11, the sensitivity of the MyMAAT-7 when compared against the MyMAAT-12, BMQ adherence level, HbA1c levels, and phosphate level changes were 84.6%, 36.3%, 62.5%, and 54.9%, respectively. The corresponding specificity values were 99.0%, 42.6%, 56.1%, and 53.8%. The false positive rate (Type I error) was 1.0%, 57.4%, 43.9%, and 46.2%, respectively, while the false negative rate (Type II error) was 15.4%, 63.7%, 37.5%, and 45.1%. The PPV and NPV were 99.2% and 82.3% with the MyMAAT-12; 27.0% and 53.2% with the BMQ adherence level; 45.5% and 71.9% with HbA1c levels; and 60.0% and 48.6% with phosphate level changes.

**Table 11:** Sensitivity and specificity test for MyMAAT-7 scale.

MyMAAT-7 classification (n = 246)	MyMAAT-12 Classification of Poor to moderate adherence	MyMAAT-12 Classification of Good adherence	Positive and negative predictive value
Poor to moderate adherence (Score <32)	121	1	Positive PV = 99.2%
Good adherence (Score ≥32)	22	102	Negative PV = 82.3%
Sensitivity and specificity	Sensitivity = 84.6%		Specificity = 99.0%
**MyMAAT-7 classification** **(n = 246)**	**BMQ (Ambivalent, Indifferent, and Skeptical)**	**BMQ (Accepting group)**	**Positive and negative predictive value**
Poor to moderate adherence (Score <54)	33	89	Positive PV = 27.0%
Good adherence (Score ≥54)	58	66	Negative PV = 53.2%
Sensitivity and specificity	Sensitivity = 36.3%		Specificity = 42.6%
**MyMAAT-7 classification** **(n = 130)**	**Poor control HbA1c ≥ 8%**	**Good control HbA1c < 8%**	**Positive and negative predictive value**
Poor to moderate adherence (Score <54)	30	36	Positive PV = 45.5%
Good adherence (Score ≥54)	18	46	Negative PV = 71.9%
Sensitivity and specificity	Sensitivity = 62.5%		Specificity = 56.1%
**MyMAAT-7 classification** **(n = 147)**	**Phosphate level increases**	**Phosphate level did not increase (maintained/ reduced)**	**Positive and negative predictive value**
Poor to moderate adherence (Score <54)	45	30	Positive PV = 60.0%
Good adherence (Score ≥54)	37	35	Negative PV = 48.6%
Sensitivity and specificity	Sensitivity = 54.9%		Specificity = 53.8%

## Discussion

The current study assessed medication adherence and established the psychometric properties of the MyMAAT-12 scale in patients with non-dialysis CKD. The findings indicate acceptable internal consistency, stable reliability, fair sensitivity, and validity, with significant weak to moderate associations with HbA1c% and phosphate levels changes. This study also offers the first empirical insight into adapting the MyMAAT-12 scale into a shorter version, the MyMAAT-7, which demonstrated satisfactory reliability and validity.

### Medication belief and adherence

Our study reported that most participants with non-dialysis CKD were non-compliant with their medications. This finding aligns with previous studies, which observed similar rates of non-adherence in CKD populations [10,26,33]. The complexity and number of medications prescribed are well-documented factors influencing adherence [10,19,26]. However, our study did not observe any significant association between medication adherence and either the number of medications prescribed or the stages of CKD. This finding contrasts with some previous studies that reported a link between a higher medication burden or disease severity and poorer adherence. The discrepancy may highlight the multifactorial nature of medication adherence, particularly in CKD populations, where factors beyond clinical parameters—such as personal beliefs about medication, perceived social support, and health literacy—play a more dominant role in influencing patient behaviour [9,10,21,33]. In the Malaysian context, cultural attitudes toward medications and reliance on traditional remedies may further modulate adherence behaviours [9,10]. These multifaceted influences may explain the lack of association found in our study.

In terms of beliefs toward medication use, patients’ feedback in this study aligned with previous findings by Bai et al. (2022) [26]. The group with the highest adherence score on the MyMAAT-12 was the “accepting” group—those with high necessity beliefs and low concerns about their medications. This was followed by the “indifferent” group (low necessity, low concern), the “ambivalent” group (high necessity, high concern), and lastly, the “skeptical” group (low necessity, high concern). Similar patterns were also reported by Horne et al. (1999) and Bai et al. (2022), where medication adherence was highest among the accepting group, followed by the ambivalent, indifferent, and skeptical groups [12,26]. One possible explanation for this trend is that the MyMAAT-12 places greater emphasis on actual medication-taking behaviour (4 items) and aspects such as self-efficacy and social support (4 items), while only three items focus on beliefs related to necessity and concerns [16]. This may influence the extent to which beliefs are reflected in adherence scores.

### Reliability of MyMAAT-12 scale among patients with CKD

The MyMAAT-12 demonstrated strong reliability for assessing medication adherence among non-dialysis CKD patients, as reflected by an excellent Cronbach’s alpha of 0.90. This high internal consistency suggests that the scale items are well-correlated and consistently measure the underlying construct of adherence. The observed reliability is comparable to, and even exceeds, that of other widely used tools in similar populations, such as the BMQ-Necessity (α = 0.83) and BMQ-Concern (α = 0.82) reported by Bai et al. (2022) [26]. Additionally, MyMAAT-12 outperformed other adherence tools validated in Malaysian settings, including the MALMAS (α = 0.57), BMQ (α = 0.62–0.76), and MMAS (α = 0.68) [28,35,36], highlighting its potential suitability and cultural relevance for local use.

### Hypothesis testing validity of MyMAAT-12 scale

The observed convergent and known-groups validity of the MyMAAT-12 against the BMQ-Specific scale provides preliminary support for its use in assessing medication adherence among patients with CKD. The choice of BMQ-Specific as a comparator was appropriate, given its robust application in chronic disease populations. However, modest sensitivity and specificity values suggest that the MyMAAT-12 may capture different dimensions of adherence compared to belief-based tools like the BMQ. The significant correlation with the BMQ-Concern score—but not with the BMQ-Necessity score—offers further insight into this distinction. It is plausible that concerns about medication, such as fears of side effects or long-term dependence, may have a more immediate impact on behavioral adherence than perceived necessity, particularly in patients managing asymptomatic or slowly progressing conditions like CKD [34]. This may explain why the MyMAAT-12, which includes items addressing practical barriers, confidence in self-management, and social support, aligns more closely with patient concerns. Furthermore, the discrepancy between necessity beliefs and actual behaviour may also reflect the dynamic and sometimes contradictory nature of adherence. For instance, a patient may intellectually believe a medication is necessary but still struggle with taking it consistently due to lifestyle constraints, forgetfulness, or emotional factors—domains more directly assessed by the MyMAAT-12 [16]. These findings highlight the importance of using multi-dimensional adherence tools that go beyond cognitive beliefs to include behavioral and contextual factors.

Besides, the MyMAAT-12 demonstrated satisfactory known-groups validity when tested against clinical parameters such as HbA1c% and phosphate level changes. In diabetic CKD patients, the tool showed a sensitivity of 72.9% and a specificity of 46.3% for detecting poor glycemic control. These values are comparable to prior findings for MALMAS (sensitivity 88.9%, specificity 29.6%) and MMAS (sensitivity 77.6%, specificity 45.4%) in similar populations [28,35,36]. The MyMAAT-12 also performed reasonably well when tested against phosphate level trends. According to clinical practice guidelines, maintaining serum phosphate levels within the normal range is essential in CKD management [1,37]. Therefore, an increasing trend in phosphate levels may reflect non-adherence, whether to phosphate binders or dietary restrictions. However, both HbA1c and phosphate levels are influenced by various factors beyond medication adherence, such as physical activity, dietary habits, and disease progression [38]. The relatively high sensitivity but lower specificity of the MyMAAT-12 may also be due to under-reporting of non-adherence, a known limitation in self-reported measures, especially when patients respond in the presence of healthcare providers [36]. These findings highlight the need for cautious interpretation of adherence tools and reinforce the value of combining patient-reported outcomes with clinical and behavioral indicators.

When evaluated against established referent standards—including the BMQ, HbA1c levels, and phosphate level changes—the MyMAAT-12 showed a lower PPV, ranging between 31.5% and 63.6%, compared to its NPV, which ranged from 55.3% to 74.5%. These findings mirror those reported in the original validation of the scale among diabetic patients [16]. This suggests that the MyMAAT-12 is more effective at identifying patients who are truly adherent (i.e., correctly classified as adherent when they report good adherence) than those who are truly non-adherent. In practical terms, patients who are classified as adherent by the tool (negative test) are more likely to be truly adherent (true negatives), while those flagged as non-adherent (positive test) may include a larger proportion of false positives. Although this reduces the tool’s precision in identifying true non-adherence, it helps minimize the risk of overlooking adherent patients (i.e., fewer false negatives). In a clinical context such as CKD, where the consequences of missed non-adherence can be severe, a tool with higher negative predictive value still holds value—particularly for ruling out adherence issues and guiding further clinical review or patient counselling.

### Reduction analysis of MyMAAT-12

This study represents the first attempt to reduce the number of items in the MyMAAT-12 scale, resulting in the development of the shorter MyMAAT-7. During the item-reduction process, Items 4, 5, and 8 were removed due to lower extracted communalities (<0.5). Upon content review, Item 4 (“being late or missing an appointment to receive a medication supply”) and Item 5 (“having excess medication supply”) were considered less reflective of true medication adherence behaviours, particularly within the context of government healthcare settings where pharmacists may provide patients with additional medication beyond scheduled appointments. Item 8, which addresses “fear of side effects,” was also excluded, as it may not adequately capture the broader range of patient concerns related to medication use. In addition, Items 1 and 12 were removed due to conceptual redundancy. Item 1 and Item 7 both focus on non-adherent behaviour (“failing to take medications”), while Item 12 and Item 9 both relate to the influence of social support on adherence. By removing overlapping items, the revised scale maintains its core domain while enhancing efficiency. Despite the reduction in items, the MyMAAT-7 demonstrated satisfactory psychometric properties. Internal consistency remained strong, with a Cronbach’s alpha of 0.87—only slightly lower than the original scale (α = 0.90). The shortened scale also showed acceptable sensitivity and specificity when tested against clinical parameters, albeit slightly reduced compared to the full MyMAAT-12. These findings suggest that the MyMAAT-7 offers a more concise yet reliable alternative for assessing medication adherence. In clinical practice, both the MyMAAT-12 and MyMAAT-7 can be integrated into routine pharmacist-led medication adherence assessments to support real-time monitoring and targeted interventions. The brevity of MyMAAT-7 makes it particularly suitable for high-volume public healthcare settings. Further validation in larger and more diverse populations is recommended to confirm generalizability and utility across different healthcare settings. Future studies should also investigate predictive validity by linking adherence scores to long-term clinical outcomes, using longitudinal data and multivariate models to account for potential confounders.

## Limitations

This study has several limitations that should be acknowledged. First, the cross-sectional design limits the ability to capture changes in medication adherence behaviour over time. Although test-retest reliability over a two-week period supported short-term stability, future longitudinal studies are needed to assess long-term consistency and predictive validity. Second, the use of a self-administered questionnaire introduces the potential for response bias. Despite assurances of anonymity and efforts to reduce social desirability bias, adherence may have been overestimated due to self-reporting. Third, the study sample may not fully represent the broader population of patients with chronic diseases, limiting the generalizability of the findings. Moreover, correlation and diagnostic accuracy statistics do not establish causal relationships, and the observed associations may differ across populations or settings. Fourth, as this study focused on instrument validation using EFA, it did not model predictive relationships, examine the influence of potential confounders, or explore predictive associations. Finally, while clinical parameters such as HbA1c% and phosphate levels were used as indirect adherence indicators, these can be influenced by non-adherence–unrelated factors such as dietary intake, exercise, and disease progression. Future research should explore the validity of the MyMAAT-12 and MyMAAT-7 in other populations and settings, using longitudinal designs.

## Conclusions

This study successfully evaluated the psychometric properties of the MyMAAT-12 among patients with non-dialysis CKD. The scale demonstrated good internal consistency, stability, and validity with meaningful associations with clinical markers such as HbA1c% and phosphate levels. Although its sensitivity and specificity were moderate, the MyMAAT-12 performed comparably to other established adherence tools. Additionally, the development of the shorter MyMAAT-7 offers a more concise alternative, while retaining acceptable reliability and validity. Further research is needed to validate both versions in broader and more diverse populations, using longitudinal designs. Overall, these findings support the use of MyMAAT scales as valuable tools for measuring medication adherence in CKD care.

## Supporting information

S1 File MyMAAT-CKD patient data. (XLSX)
